# Bioreactor Technology for Medicinal Plant In Vitro Cultures: Systems, Applications, and Future Perspectives

**DOI:** 10.3390/biology15131025

**Published:** 2026-06-27

**Authors:** Shuang Zhang, Meibing Ma, Ying Liu, Heng Jiang, Jie Gao, Quan Yang, Kunhua Wei

**Affiliations:** 1 Key Laboratory of State Administration of Traditional Chinese Medicine for Production & Development of Cantonese Medicinal Materials, Guangzhou Comprehensive Experimental Station of National Industrial Technology System for Chinese Materia Medica, Guangdong Engineering Research Center of Good Agricultural Practice & Comprehensive Development for Cantonese Medicinal Materials, School of Chinese Materia Medica, Guangdong Pharmaceutical University, Guangzhou 510006, China; wenliang57321@163.com (S.Z.); 19808032056@163.com (Y.L.); jh15874556538@163.com (H.J.); gszyy1220@163.com (J.G.); 2 Traditional Chinese Medicine Resource Germplasm Bank Management Center, Yunfu 527399, China; 3 Fusui Livestock Breeding Farm, Guangxi Zhuang Autonomous Region, Chongzuo 532200, China; 18977158003@163.com

**Keywords:** bioreactor, medicinal plants, in vitro culture, secondary metabolites, industrialization

## Abstract

Bioreactor technology for medicinal plants provides a controllable platform for the conservation of rare and endangered resources, the production of high-value-added active ingredients, and green manufacturing of traditional Chinese medicine. Focusing on in vitro culture systems of medicinal plants, this article systematically reviews the application progress of stirred-tank, airlift, bubble column, wave-mixed, spray-type, temporary immersion, and photobioreactors in the culture of suspension cells, adventitious roots, hairy roots, shoots, and somatic embryos. Different from existing studies that mainly list reactor types, this review further provides a comprehensive analysis from the perspectives of physiological characteristics of the cultured objects, mass transfer and shear environment, medium and elicitor regulation, inoculation density, culture cycle, representative cases, and industrialization limitations. The results indicate that bioreactors can shorten production cycles, improve environmental controllability, and enhance product quality consistency; however, their large-scale application remains constrained by scale-up stability, metabolic fluctuations, downstream processing costs, GMP quality control, and commercial feasibility. Future research should shift from merely pursuing increased yield to integrated process development that is scalable, verifiable, low-cost, and quality-controllable.

## Simple Summary

Medicinal plants are important sources of bioactive compounds, but field cultivation often faces problems such as unstable quality, environmental variation, and overharvesting of rare species. Bioreactor technology provides a controlled and scalable approach for producing medicinal plant materials under standardized and sterile conditions. This review summarizes the main characteristics of different bioreactor types and their applications in suspension cell and adventitious root cultures. It also discusses key factors affecting biomass growth and metabolite production, providing guidance for medicinal plant conservation and green manufacturing.

## 1. Introduction

A bioreactor is an integrated system that supplies cells, tissues, or intact organs with a finely controllable microenvironment, thereby enabling specific biochemical conversions or supporting predefined physiological functions. Originally established for microbial fermentation and animal cell amplification, this technology has now been extended to plant biotechnology. It is increasingly recognized as a major approach for achieving large-scale production of plant bioactive compounds [1].

Medicinal plants are an important source of natural medicines, traditional drugs, and a variety of high-value-added active ingredients, playing an irreplaceable role in maintaining human health, developing plant-derived pharmaceuticals, and advancing the natural products industry [2,3]. As the population grows, health-conscious consumption increases, and the modernization of traditional Chinese medicine accelerates, the market demand for high-quality medicinal plant resources and plant-derived active ingredients continues to increase. Previous studies have indicated that population growth and the rising demand for Chinese medicinal materials are important factors driving changes in traditional Chinese medicine resources. Wild medicinal plant resources can no longer meet social demand, and the source of medicinal materials is gradually shifting from wild resources to artificial cultivation [4]. However, many medicinal plants have long relied on wild harvesting. Coupled with habitat alteration, resource overexploitation, and expanding market demand, these factors have led to a significant reduction in some wild medicinal plant resources, which are now even at risk of endangerment [5,6]. Meanwhile, although artificial cultivation has alleviated the contradiction between resource supply and demand to some extent, cultivated medicinal plants still face challenges such as long growth cycles, low propagation efficiency, continuous cropping obstacles, pests and diseases, pesticide residues, poor ecological adaptability, and fluctuations in active ingredient content. These issues affect the quality, stability and batch-to-batch consistency of medicinal plant raw materials [4,7,8,9]. Particularly for rare and endangered medicinal species that are slow-growing, scarce in wild resources, or dependent on endophytic symbiosis, the issue of sustainable supply is especially prominent [7,10,11]. In the development of modern medicinal plant resources, the traditional agricultural model that simply pursues high yield can no longer meet the demands for high quality, standardization, and sustainable development. Green agriculture and ecological agriculture emphasize resource efficiency, environmental friendliness, quality safety, and process controllability, with the core objective of achieving a coordinated balance among agricultural product safety, ecological security, resource security, and comprehensive economic benefits [12,13]. This is highly consistent with the goals of modern Chinese medicine resource protection, quality improvement of medicinal materials, and stable supply of plant-derived active ingredients. Therefore, establishing a novel technological system that integrates resource protection, quality regulation, and potential for large-scale production has become an important direction for the sustainable utilization of medicinal plant resources [2,7,14].

The cultivation of plant cells or organs (e.g., suspension cells and adventitious roots) in bioreactor systems provides a stable and efficient in vitro production platform, thereby presenting a promising and substantiated solution to the sustainable supply challenges of rare and endangered medicinal plants [2]. Moreover, classical cultivation methods such as shaking flasks and solid-state substrates suffer from cumbersome operations, low amplification efficiency, and imprecise control of environmental factors, severely limiting industrial application [7,15]. Bioreactors, based on a closed and controllable culture system, can monitor and regulate key process parameters such as temperature, pH, dissolved oxygen, aeration, agitation, medium composition, and nutrient supply under sterile conditions, thereby providing a relatively stable growth environment for plant cell, tissue, or organ culture [16,17]. In the suspension culture of medicinal plant cells, adventitious root and hairy root cultures, suitable reactor types, medium composition, aeration methods, feeding strategies and induction regulation can promote biomass accumulation and increase the yield or specific productivity of target metabolites such as phenolics, flavonoids, triterpenoid saponins and sapogenins. Meanwhile, closed, sterile and repeatable controlled culture conditions help reduce the risk of exogenous contamination and improve the standardization, batch stability and quality consistency of plant-derived pharmaceutical raw materials [18,19,20,21].

To address the physiological characteristics of different medicinal plant tissues, bioreactor systems of appropriate scale must be developed, with dynamic regulation of key parameters such as dissolved oxygen, pH, and shear stress. This approach alleviates resource scarcity, replaces wild harvesting, and underpins the standardization and green manufacturing of traditional Chinese medicine. This strategy enables the continuous supply of active ingredients and the large-scale production of secondary metabolites [22]. This paper aims to review the current state of research in this field and to outline its future development trends.

## 2. Methodology

This review focuses on bioreactor technologies for the in vitro culture of medicinal plants and was prepared through a structured, targeted literature search combined with an analysis of representative studies. The literature search aimed to collect published studies related to bioreactor design, plant cell and organ culture systems, biomass production, biosynthesis of bioactive or marker metabolites, process optimization, and scale-up strategies.

The literature search was conducted using the following academic databases: Web of Science, Scopus, PubMed, Science Direct, Springer Link, and Google Scholar. Chinese-language articles were also retrieved from the China National Knowledge Infrastructure (CNKI). Based on the scope of this review, combinations of keywords were used, including: bioreactor, plant cell culture, plant tissue culture, medicinal plant, bioactive compounds, marker metabolites, secondary metabolites, suspension cell culture, adventitious root, hairy root, somatic embryo, shoot culture, temporary immersion system, airlift bioreactor, bubble column bioreactor, stirred-tank bioreactor, mist bioreactor, wave-mixed bioreactor, photobioreactor, elicitation, methyl jasmonate (MeJA), process scale-up, good manufacturing practice (GMP), industrial production, and commercial application.

The literature search primarily covered the period from 2000 to 2025, during which major advances in single-use bioreactors, temporary immersion bioreactors, and industrial applications have been reported. Selected earlier publications were also included when they were foundational to understanding reactor principles, oxygen transfer, mixing and mass transfer, scale-up mechanisms, or when they reported successful early cases of plant cell, tissue, or organ culture in bioreactors. Therefore, the reference list includes both classic foundational works and recent advances, ensuring the completeness of the research context and the timeliness of the review.

## 3. Types and Engineering Characteristics of Medicinal Plant Bioreactor Cultivation Systems

### 3.1. Overview of Bioreactor Technology

A bioreactor refers to a system that provides a microenvironment with controllable multidimensional parameters—such as temperature, pH, dissolved oxygen, and shear stress—for biological units including enzymes, cells, tissues, and organs. This enables the continuous operation of specific biochemical reactions or the maintenance of target metabolic pathways. Primarily applied in pharmaceutical production, organic pollutant degradation, fermentation for concentrated fruit juices and jams, and alcoholic beverage production, its scope has recently expanded into the field of in vitro cultivation of medicinal plants [23]. The distinct characteristics of plant cells compared to animal cells and microorganisms necessitate tailored structural and operational designs for bioreactor systems [24]. Plant cells are typically large in size and tend to form cell aggregates of varying scales in liquid culture; therefore, their growth and metabolic processes are sensitive to the efficiency of mixing, dissolved oxygen, and nutrient transfer. Previous studies have indicated that mixing and oxygen transfer are critical issues in process optimization for high-density plant cell suspension cultures. Moreover, larger plant tissue or cell aggregates are prone to mass transfer limitations at the medium–tissue interface, which can result in insufficient oxygen and nutrient supply within the interior of the aggregates [25,26]. Meanwhile, plant cell suspension cultures often exhibit characteristics such as cell aggregation, increased viscosity, foam formation, and shear sensitivity. Consequently, in stirred or bubble column bioreactors, it is necessary to achieve sufficient mixing to maintain nutrient and oxygen supply while keeping hydrodynamic shear within a range that does not cause cell damage [27,28]. For adventitious root and hairy root cultures, the root systems tend to form entangled clumps in liquid environments, which in turn affects aeration, nutrient transfer, sampling homogeneity, and process scale-up. For example, in the culture of *Oplopanax elatus* adventitious roots in a 5 L fed-batch bioreactor, researchers need to control the inoculation amount, aeration rate, culture temperature, and feeding strategy to sustain root growth and flavonoid accumulation [20]. Furthermore, embryoids, bud primordia, and excised buds are sensitive to liquid contact duration, gas exchange, and culture humidity. Continuous immersion or excessive humidity can readily induce vitrification, suffocation, or morphological abnormalities. In contrast, temporary immersion bioreactors can mitigate such risks to some extent by providing periodic immersion and gas exchange [29,30]. Therefore, the selection of a bioreactor system should not solely consider scale-up efficiency but must also be comprehensively optimized by integrating the morphological structure, physiological sensitivity, and engineering mass transfer conditions of the cultured object.

Bioreactors create optimal conditions for plant tissue growth and metabolite synthesis by regulating parameters such as temperature, pH, dissolved oxygen, mixing intensity, and nutrient supply. Based on the culture chamber environment, bioreactors may be broadly categorized into four types: liquid-phase, gas-phase, mixed bioreactors, and temporary immersion bioreactors (TIB) [31]. Liquid-phase bioreactors may be further subdivided into three subgroups: Stirred tank bioreactors (STRS), Airlift bioreactors, and Recirculating or convective bioreactors, depending on the type of energy input employed for mixing [32].

### 3.2. Major Types of Bioreactors and Their Applicability

Based on mixing and oxygen transfer mechanisms, bioreactors suitable for medicinal plant cultivation can be categorized into the following types.

#### 3.2.1. Stirred Tank Bioreactor

A stirred tank bioreactor (STR) is a widely used type of reaction device in the field of bioengineering and biopharmaceuticals [33], which is commonly used in the processes of microbial fermentation, cell culture and enzyme catalysis. The rotating paddles disperse the gas phase into fine bubbles, which increases the oxygen mass transfer coefficient; electrodes and sensors are embedded in the sidewalls, so that the temperature, dissolved oxygen, and foam level are transmitted back in real time, and the control commands are issued in milliseconds [34]. However, the high shear forces generated at the end of the stirring paddles are prone to cause mechanical damage to plant cells and tissues, which incidentally suspends the synthesis of secondary metabolites [35]. Therefore, STR is more suitable for shear-tolerant plant suspension cell culture. For example, during the cultivation of *Artemisia annua* hairy roots, by optimizing stirring conditions, the yield of artemisinin obtained in STR can be significantly higher than that in traditional shaking flask cultures [36].

#### 3.2.2. Wave-Mixed Bioreactor

The wave-mixed bioreactor utilizes pre-sterilized membrane culture bags (such as Cytiva Cellbag) [37,38], which are shaken to create waves of liquid inside the bag for gentle mixing and oxygenation. Its closed structure and design without mechanical agitation effectively avoids contamination and reduces shear injury. However, the system also suffers from difficulties in online monitoring, possible reduction in mixing and oxygenation efficiency after amplification, and cost and environmental problems of disposable consumables [1]. Therefore, it is particularly suitable for culturing plant material that is sensitive to shear forces and requires high aseptic conditions.

For example, when hairy root cultures of *Panax ginseng* and *Rindera graeca* were cultivated in wave bioreactors, both biomass and saponin production were significantly higher than those obtained in traditional shake-flask cultures [39,40]. Moreover, Gitzinger et al. [41] demonstrated that when transgenic moss protoplasts were microencapsulated and cultured in a wave-mixed bioreactor, the yield of the target recombinant protein was comparable to that achieved in stirred-tank cultures, further validating the potential applicability of wave bioreactors in plant recombinant protein production.

#### 3.2.3. Pneumatic Bioreactors

Pneumatic bioreactors (BCBs, including Airlift and Bubble Column configurations) circulate liquids driven by the lift force generated by the passage of gas, and have no mechanical stirring structure with low shear forces. Advantages are simple structure, low manufacturing cost, easy to clean and sterilize [42]. Disadvantages are that the mixing efficiency decreases as the viscosity of the culture solution increases, poor mixing of dense or agglomerative cultures, and the presence of a pronounced dead zone in the flow pattern [43]. This type of bioreactor is suitable for organ cultures that are highly sensitive to shear stress. For instance, Ming et al. have found that alkaloid yields were significantly higher in *Scopolia parviflora* adventitious root cultures than in shake flask cultures [44].

#### 3.2.4. Sprinkle or Spray Bioreactor

The sprinkle or spray bioreactor (SBR) belongs to the gas-phase culture system, and its logic of operation is to tear the liquid culture medium into aerosols with particle sizes of 1–30 μm with the help of high-frequency microporous or dual-fluidic nozzles, and then uniformly settle on the surface of cultures on the stationary support in a set cycle, and the whole process maintains the dynamic renewal of the substrate-gas interface. As a result, plant tissues are almost entirely in the gas phase, with the oxygen partial pressure approaching the ambient saturation concentration, and the liquid-phase shear stress is compressed, which is particularly friendly to vulnerable meristematic zones [45]. However, the spatial nonuniformity of the atomization field often results in a 2–3 fold variation in droplet flux, with subsequent fluctuations in local nutrient concentrations; furthermore, in order to maintain a sterile aerosol circuit, the system needs to be integrated with sterilizing filters, a reflux condensation section, and autoclavable nozzles, making it a relatively complex equipment structure [46]. Because of these properties, these reactors are particularly suitable for dense plant tissue cultures that require efficient oxygen exchange. Batch cultures of *Arachis hypogaea* hairy roots in a 1 L bioreactor achieved a specific growth rate of μ = 0.173 d^−1^ and a biomass concentration of 12.75 g DW/L, compared with μ = 0.166 d^−1^ and 11.10 g DW/L in shake-flask cultures under the same medium composition. At the 1 L scale, the spray bioreactor outperformed shake-flask culture, increasing the growth rate by about 4% and the dry-weight biomass yield by about 15%. When the reactor was scaled up to 20 L, the results of μ = 0.147 d^−1^ and 7.77 g DW/L were successfully achieved by increasing the nutrient flow rate instead of simply extending the spray time. Moreover, the study validated the predictive accuracy of the deposition model for droplet capture [47].

#### 3.2.5. Temporary Immersion Bioreactors

The temporary immersion bioreactors (TIB) are designed to inject culture fluid in a programmed pulsed manner, so that the explants are completely submerged within seconds to minutes, and then rapidly drained, forcing them to return to the gas phase again. This cyclic submergence mechanism cleverly combines the efficient nutrient supply of liquid-phase culture with the sufficient gas exchange of gas-phase culture, which helps to alleviate the problems of tissue hypoxia and vitrification that are easily caused by prolonged total submergence culture [48]. However, the proliferation efficiency is highly sensitive to the frequency of submergence, the duration of a single session and its coupling mode; the same set of parameters transferred to different species or even different explant types of the same species often requires recalibration [49]. In the two major fields of micropropagation and somatic embryogenesis, the reactor has been successfully used, for example, a temporary immersion culture test was conducted on *Dendrobium nobile* Lindl. plantlets, and it was found that the value-added multiplicity and alkaloid content were the highest when the submerged time was 5 min/8 h, the inoculum volume was 10 g/L, and the concentration of sucrose was 20 g/L. Meanwhile, the frequency of submerging was the key to affecting the value-added of *D. nobile*, and it could reduce the consumption of sucrose [50].

#### 3.2.6. Balloon-Type Bubble Bioreactor

The balloon-type bubble bioreactor (BTBB) is a special configuration of the air-lift reactor, its cavity at the equator suddenly expanded into a thin-walled crown, where the bubble group decelerated, collision and redispersion, the probability of fusion has been amplified, and the rise path has been homogenized; after the high diameter ratio has been compressed, the overall flow field tends to be low shear, low turbulence, and the energy dissipated in the macroscopic circulation rather than local vortex, the living cells can maintain the membrane integrity in the mild hydrodynamic impulse. The membrane integrity is maintained and the mixing time is shortened [51,52]. However, the spherical crown molds, reducer flanges and highly polished internal surfaces push the processing man-hours up, with a single unit costing about 1.4–1.6 times that of a straight tank of the same volume. This premium was partially offset in scale-up experiments, such as in the high-density culture of *Panax ginseng* adventitious roots, where the use of a balloon-type bubble bioreactor in *Panax ginseng* adventitious roots production yielded significant results, with maximum yields of approximately 500 g and 2.2 kg at 42 d in 5 and 20 L bioreactors, inoculated with 60 g and 240 g, respectively. Choi et al. increased the total amount of biomass produced by cutting twice during incubation, and the amount of root biomass in a 20 L BTBB was 2.8 kg at harvest; inoculation of 500 g fresh weight adventitious roots into a 500 L balloon bubble bioreactor, cut at 4 and 6 weeks after inoculation, produced about 74.8 kg of roots, which exhibited similar levels of ginsenoside content compared to field cultivation [53].

#### 3.2.7. Photobioreactors and Light-Regulated Culture Systems

A photobioreactor (PBR) is a culture system that uses light as the core regulatory factor. It provides a controllable environment for photoautotrophic or light-responsive cultures by adjusting parameters such as light quality, intensity, photoperiod, CO_2_ supply, and temperature [54]. Traditionally, this type of reactor has been primarily used for the cultivation of photosynthetic organisms such as microalgae, cyanobacteria, and mosses [55,56]. However, in recent years, it has also been introduced into plant cell culture systems to regulate light signal-mediated secondary metabolite biosynthesis. Previous reviews have indicated that light quality, intensity, and photoperiod can influence the biosynthesis of plant secondary metabolites such as polyphenols, flavonoids, terpenoids, and alkaloids. Furthermore, different light sources, including LEDs, ultraviolet (UV) light, and fluorescent light, can serve as abiotic elicitors in in vitro culture systems [57]. For example, when cocoa cells were cultured in a 7.5 L stirred-tank photobioreactor and exposed to white and blue LED light, the accumulation of proanthocyanidins and the expression of genes related to flavonoid biosynthesis showed significant changes, indicating that light can serve as an important environmental factor for regulating phenolic compound accumulation in large-scale plant cell cultures [58]. Compared with traditional dark or low-light culture systems, photobioreactors facilitate the study of the relationships among light signaling, ROS signaling, and secondary metabolic pathways [59]. However, their application in the industrial-scale cultivation of medicinal plants remains limited by issues such as insufficient light penetration, shading by cell aggregates, inhomogeneous light field distribution after reactor scale-up, and high energy consumption of artificial light sources [60,61].

### 3.3. Bioreactor Type Selection

Based on the aforementioned engineering characteristics of the various types of bioreactors, their selection needs to take into account the physiological properties of the plant tissues and the synthesis needs of the products. Table 1 below systematically compares the core features of each bioreactor.

In summary, the selection of bioreactor should first consider the physiological and mechanical properties of the culture object itself, hairy roots have extremely low shear threshold, air-lift or wave reactor borrows bubble pulsation or liquid surface oscillation to complete the oxygen delivery, which precisely avoids the high shear zone of the vane and paddle; buds and somatic embryos need to be intermittently submerged with the cyclic updating of the gas–liquid interface, and the TIS or nebulized bed thus becomes an optimal solution for the gas exchange efficiency; suspension cells can tolerate eddy shear due to their thick cell walls and small aggregate size, and the STR is coupled with a three-loop speed-dissolved oxygen–pH coupling. Because of the thick cell wall and small aggregate size, suspension cells can withstand the vortex shear of Rushton paddles, and STR still occupies the core position of large-scale amplification with the precision of the three-loop coupling of rotational speed, dissolved oxygen and pH. In practice, the final decision needs to be made by combining training objectives, costs and potential for scaling up [65].

## 4. Bioreactor Cultivation Process Parameters

### 4.1. Regulation of Physical Parameters and Their Impact

In bioreactor culture systems, physical parameters directly affect plant cell and tissue growth and secondary metabolism by influencing the mass transfer efficiency and hydrodynamic environment. Among them, agitation and aeration together determine the rate of oxygen transfer and shear force in the system.

#### 4.1.1. Stirring and Shear Force

The mixing speed directly affects the mixing and mass transfer efficiency of oxygen and nutrients, firstly by changing the shear strength of the flow field, which in turn alters the cell growth state and metabolite synthesis capacity. For stirred tank reactors, the paddle diameter D is coupled to the rotational speed N in cubic-fifth power, with power per unit volume P/V ≈ N^3^D^5^; the magnitude of the power input further determines the volumetric oxygen transfer coefficient (kLa), and thus an appropriate increase in the rotational speed can help to enhance the oxygen supply to avoid the growth of cultures being limited due to insufficient dissolved oxygen [66]. However, excessive power paddle tip linear velocities jump linearly, and the gradient of the shear peak can instantly tear the plant cell mass periphery, with segmental rupture of hairy roots, intracellular Ca^2+^ pulses triggering programmed death cascades, and the consequent silencing of secondary metabolic pathways [67,68].Therefore, the essence of mixing design is to find the narrowest safety belt between the oxygen supply curve and the damage threshold: prioritize scaling up the paddle diameter rather than the rotational speed, and keep the tip speed down with a ‘big paddle, low rotational speed’ strategy while maintaining a constant P/V; at the same time, replace the paddle with an airfoil or a marine paddle, and let the axial circulation replace the radial jet, so that the energy is dissipated in the macroscopic vortex rather than microscopic shear [69]. It is also possible to calibrate kLa online and set it as a feedback variable, refusing to blindly chase high speeds [70]. For example, in fungal fermentation, researchers have found that *Ganoderma multistipitatum* strains achieve the highest laccase yields and high sterilization densities at 150 rpm, whereas excessive speeds (e.g., 200 rpm) result in structural damage to the mycelium and reduced yields [71].

#### 4.1.2. Aeration and Mass Transfer

Aeration rate is an important control parameter in bioreactor operation. Changes in aeration intensity affect gas–liquid mixing, oxygen transfer efficiency, and dissolved oxygen levels in the culture system. As the superficial gas velocity or volume of gas per volume of liquid per minute (vvm) increases, gas holdup and the gas–liquid interfacial area generally increase, thereby enhancing the volumetric oxygen transfer coefficient (kLa) and providing a more adequate oxygen supply for plant cell, tissue, or organ culture [25,30]. However, excessively high aeration rates are not always beneficial to the culture process. On the one hand, direct sparging and vigorous aeration can increase bubble-cell contact, bubble coalescence and breakup, as well as local hydrodynamic stresses generated by bubble rupture at the liquid surface, thereby leading to reduced membrane integrity, cell lysis, or decreased regenerative capacity of plant suspension cells [27,72,73]. On the other hand, continuous aeration can also promote foam formation and wall growth, leading to fluctuations in liquid level, a reduction in effective culture volume, increased sampling errors, and a higher risk of contamination and operational failure. In a stirred-tank culture of *Morinda elliptica* suspension cells, the researchers found that bubble-free aeration completely eliminated the foaming problem, indicating that bubble behavior is an important factor affecting the stable operation of plant cell bioreactors [74]. For three-dimensional structured tissues such as adventitious roots and hairy roots, aeration rate and gas distribution pattern also affect the aeration, nutrient transfer, and mechanical damage degree of root clumps. In the bioreactor culture of *Panax ginseng* adventitious roots, gradually increasing the aeration rate from 0.05 vvm to 0.3 vvm was found to be more beneficial for root biomass and ginsenoside accumulation than constant aeration. Meanwhile, an aeration rate of 0.1 vvm has been reported as a suitable condition for *Echinacea purpurea* adventitious root culture [75,76]. Therefore, optimization of the aeration rate requires a balance among enhanced oxygen transfer, control of shear damage, foam suppression, and stability of tissue morphology.

During actual cultivation processes, the appropriate aeration range must be optimized according to the specific biological system and reactor type. For example, it was found that an aeration of 0.15 vvm was most favorable for the accumulation of biomass and flavonoid constituents in adventitious root cultures of *Gynura procumbens*, a flat-lying chrysanthemum, and that too much (≥0.20 vvm) or too little aeration had an inhibitory effect [77]. In *Rhodiola crenulata* callus cultures, conditions at 0.1 vvm were optimal for biomass growth, whereas synthesis of *Rhodiola rosea* glycosides tended to increase with increasing aeration [78]. Regarding *Catharanthus roseus* hairy root cultures, ajmalicine yield was highest at the lowest aeration (0.3 vvm) and instead decreased when elevated to 0.8 vvm, which was mainly due to physiological damage to the hairy roots caused by the shear force generated by the high aeration rate [79].

#### 4.1.3. Other Physical Parameters

In addition to agitation and aeration, temperature and light are also important physical parameters affecting plant in vitro culture. Temperature primarily influences culture growth and secondary metabolite accumulation by modulating enzymatic reaction rates, membrane fluidity, and cellular metabolic activity. The optimal temperature range generally depends on the species’ origin and the characteristics of the culture system [1,22]. Light is not only an energy source for photosynthetic cultures but also an important signaling factor. It regulates morphogenesis, chloroplast development, antioxidant responses, and metabolic pathways such as those of phenylpropanoids, flavonoids, and anthocyanins via photoreceptor-mediated signaling pathways [80,81]. Light intensity exerts dose-dependent effects on cultures: an appropriate light intensity promotes photosynthetic carbon assimilation and biomass accumulation, whereas insufficient light may restrict morphological development and metabolic synthesis, and excessively high light intensity can induce photoinhibition, reactive oxygen species accumulation, and growth suppression [80,82]. Light quality also affects culture performance; different wavelengths regulate growth and metabolism via distinct photoreceptors. Blue light is often associated with chloroplast development, antioxidant responses, and flavonoid accumulation, whereas red and far-red light are more involved in photomorphogenesis and developmental regulation [80,81,83]. Therefore, light conditions should be optimized according to the culture type, target product, and culture stage. However, achieving uniform and efficient light regulation in bioreactors remains challenging. As the culture scale increases, issues such as light attenuation, shading by cells or tissues, local heat accumulation, and uneven light distribution tend to occur within the reactor—phenomena that are particularly pronounced in high-density suspension cell, adventitious root, and hairy root cultures [1,43]. Therefore, many industrial-scale plant cell cultures currently still mainly adopt heterotrophic or low-light culture modes [84]. In contrast, light regulation is better suited for photoautotrophic micropropagation, green tissue culture, shoot and somatic embryo culture, as well as specific culture systems targeting light-responsive metabolite accumulation [22,31,81]. In the future, a control strategy that matches light intensity and light quality with the culture stage should be established by integrating LED light sources, light-transmitting reactor structures, and online temperature control.

### 4.2. Regulation of Chemical Parameters and Their Influences

The chemical composition of the culture medium directly affects plant cell and tissue growth and secondary metabolites. It involves not only nutrient supply but also osmotic pressure regulation, signaling and stress response. Classic basal medium systems, represented by MS medium and B5 medium, along with subsequent optimization studies, have demonstrated that the composition and concentrations of mineral elements, carbon sources, vitamins, organic additives, and plant growth regulators are fundamental factors determining the growth status, differentiation capacity, and metabolic characteristics of in vitro cultures [85,86,87]. In plant cell and organ cultures, medium optimization, nutrient feeding, precursor addition, and elicitor treatment are commonly used to promote biomass accumulation and the synthesis of target secondary metabolites [82,88,89]. Therefore, medium composition and elicitation treatments are not routine culture conditions independent of the bioreactor, but rather important process variables that determine reactor operating efficiency, the physiological status of the culture, and the accumulation of target products [1,88]. In shake flasks or solid culture systems, medium composition typically affects local growth, differentiation, and metabolic accumulation. In bioreactors, however, nutrient salt concentrations, carbon source supply, plant growth regulators, and elicitors also interact with aeration, agitation, shear stress, liquid immersion status, dissolved oxygen levels, and mass transfer efficiency [1,90]. Previous reviews have indicated that the culture performance of plant cells or tissues in bioreactors depends not only on the culture medium itself, but also on the combined effects of reactor configuration, mixing efficiency, oxygen supply capacity, shear environment, and culture morphology [91,92]. For temporary immersion bioreactors, immersion duration, immersion frequency, aeration renewal, and the contact mode between cells and tissues and the liquid medium further influence nutrient uptake, the occurrence of vitrification, and the quality of regenerated plants [93,94]. Therefore, culture media and elicitation protocols cannot be simply transferred directly from small-scale systems to reactor systems; instead, they need to be re-optimized according to the culture type, reactor configuration, culture stage, and production objectives (Table 2).

#### 4.2.1. Medium Composition: Sucrose

Sucrose is a major carbon and energy source for cell culture and also affects the osmotic potential of the medium [95], where a lack of sugar can severely limit growth, while excessive concentrations can lead to morphological abnormalities due to high osmotic pressure and inhibition of photosynthesis. Appropriate sucrose concentration is the basis for securing cell growth and division; below the low limit, carbon starvation directly locks in biomass accumulation; above the high limit, osmotic stress may be triggered, inhibiting cellular respiration and leading to morphological developmental abnormalities. For example, in the culture of *Talinum paniculatum* hairy roots, maximum biomass was obtained under 6% sucrose conditions, while its saponin content reached its maximum at 3% to 5% concentration [96]; in *Panax ginseng* adventitious root cultures, a sucrose concentration of 30 g·L^−1^ simultaneously supported both high biomass accumulation and saponin production [97,98].

The osmoregulatory function of sucrose is particularly critical when the culture process reaches a specific window. High concentrations of sucrose can act as an effective morphogenetic signal to promote the formation and expansion of storage organs. For example, high sucrose treatment at 80-90 g·L^−1^ significantly promoted tuber weight gain and development during the induction of microtubers [99].

Short-term sucrose enrichment before ex vitro transfer may improve the developmental competence of propagules in some systems. In *Coffea arabica*, brief high-sucrose pretreatment of somatic embryos prior to transfer enhanced subsequent plant conversion and growth, suggesting that transient increases in sucrose availability, possibly together with their osmotic effects, may facilitate embryo maturation and physiological conditioning [100,101]. Beyond its developmental role, sucrose also serves as a major carbon source during reactor cultivation. In many plant in vitro cultures, sucrose may be hydrolyzed by invertases into glucose and fructose before or during uptake, although the extent and rate of this process are culture-system dependent. In batch culture, carbon availability may progressively decline or become suboptimal over time, particularly during the rapid growth phase, making feeding strategies useful for maintaining a favorable substrate level. For example, in *Artemisia annua* hairy root cultures, maintenance of a pseudo-steady-state sucrose concentration increased artemisinin accumulation to approximately 1.0 mg·g^−1^ DW [102].

In summary, the setting of sucrose concentration should take into account multiple factors such as plant species, growth stage, type of target product, and reactor operation mode to achieve the optimal balance between cell growth and secondary metabolite synthesis [103].

#### 4.2.2. Medium Composition: Basic Salt Concentration

The salt strength of the medium is directly involved in the regulation of plant cell proliferation, elongation and secondary metabolic processes through its ionic concentration and nutrient composition. The appropriate salt strength should ensure an adequate supply of carbon and nitrogen skeleton and trace elements, while keeping the total charge below the threshold for triggering stress. MS (Murashige and Skoog) [104] cultures are rich in nitrogen sources and mineral elements and are often used as a base formulation, but their higher ionic strength may trigger osmotic stress for some species. For example, in the cultivation of *Ocimum basilicum*, higher salt concentrations lead to a decrease in the content of its volatile components [2].

In organ culture systems such as adventitious roots and hairy roots, appropriately reducing the salt strength (for example, using 1/2 MS medium) is generally more favorable for the simultaneous accumulation of biomass and metabolites. For instance, in *Echinacea purpurea* adventitious root cultures using an airlift bioreactor, the application of 1/2 MS medium led to a concurrent increase in biomass and phenolic acid content [105]; whereas in *Salvia miltiorrhiza* adventitious root cultures, the maximum dry weight growth rate was achieved under a 3/2 MS salt concentration [106].

The setting of salt strength also needs to be comprehensively considered in combination with the volume of the reactor and the mass transfer characteristics. For instance, in a 1000 L large airlift bioreactor for *Echinacea purpurea*, a relatively high biomass can still be achieved using MS medium. In medium and small-scale systems, 1/2 MS is more conducive to maintaining a low ion load and a good aeration state in the root region [107].

In addition to total salt strength, the ratio and chemical form of specific ions also affect culture performance. In carrot suspension cells, adjusting the ratio of Ammonium nitrate (NH_4_NO_3_) to Potassium nitrate (KNO_3_) significantly increased anthocyanin production without affecting cell growth [108]; in *Nicotiana tabacum* BY-2 suspension cell cultures, the rapid depletion of phosphate was identified as a key factor limiting biomass accumulation [109].

The salt composition of different media also leads to variations in applicability. For example, in the temporary immersion culture of *Uncaria guianensis*, the performance of Woody Plant Medium (WPM) medium was superior to that of MS [110]; in *Picrorhiza kurroa* hairy root cultures, the highest biomass was obtained under full-strength Gamborg’s B-5 Medium (B5), whereas half-strength B5 was more favorable for the synthesis of the target secondary metabolite picroliv [111]. Therefore, the determination of medium salt strength must take into account total ion concentration, the ratio of key elements, species-specific physiological characteristics, and the engineering environment of the bioreactor, to maximize the overall efficiency of the culture system [107].

#### 4.2.3. Medium Composition: Growth Regulators

When the cultivation mode shifts from static or shake-flask systems to a bioreactor, the fluid shear and aeration patterns change abruptly, altering the plant cells’ hormonal perception thresholds and metabolic networks. Hormone ratios that have been validated as effective in solid or small-scale oscillatory systems almost inevitably require recalibration of concentration ranges and application rates after scale-up; otherwise, the balance between proliferation and product formation becomes immediately mismatched. Suspension cells typically maintain rapid, undifferentiated growth within a low-to-medium concentration range of 2,4-Dichlorophenoxyacetic acid (2,4-D). However, appropriate reduction or even removal of growth factors in bioreactors can effectively activate cell differentiation and related secondary metabolic pathways. For instance, in tabacum suspension cell cultures, lowering the concentration of 2,4-D significantly increased anthocyanin production to approximately 90 mg·L^−1^ [112].

Different plant species exhibit significant differences in their responses to hormone combinations. For example, in the date palm suspension cell system, the combination of 5 mg·L^−1^ 2,4-D and 2.5 mg·L^−1^ N^6^-(2-isopentenyl)adenine (2iP) can maximally promote the synthesis of phenolic and flavonoid compounds [113]. For organ-type culture systems such as hairy roots and adventitious roots, due to the self-regulatory ability of the Ri plasmid or the endogenous hormone system for root formation, no or only very low levels of exogenous auxins are usually required. In this case, the addition of auxins or gibberellins (such as Gibberellic Acid, GA_3_) may interfere with normal morphogenesis and growth processes. In contrast, methyl jasmonate, as an elicitor, bypasses the hormonal network and directly amplifies secondary metabolic pathways, showing higher induction efficiency than traditional growth regulators [114].

In the in vitro regulation of morphogenesis, the initiation of microtubers and the synchronization of somatic embryos are particularly dependent on the compatibility and timing of growth regulator application. Gibberellins usually inhibit tuber formation, and trace amounts of GA_3_ (0.1–1.0 mg·L^−1^) can completely suppress the formation of potato microtubers [115]. In contrast, substances that inhibit gibberellin synthesis or activity (such as paclobutrazol, PBZ), when used in combination with cytokinins (such as kinetin), can exert a synergistic effect—when the inhibition is released and proliferation signals are simultaneously activated—significantly increasing tuberization rates and promoting earlier maturation [116]. The type and dose of cytokinin modulate the vitrification phenomenon. In temporary submerged bioreactors, once the threshold is crossed by 6-Benzyladenine (6-BA) or Thidiazuron (TDZ), the endogenous hormone network is instantly tilted, leading to vitrification of seedlings [93]. Effective mitigation of vitrification while maintaining proliferation rates can be achieved by reducing the concentration at which it is used or by switching to a novel cytokinin (e.g., meta-topolin) [117]. In somatic embryogenesis systems, abscisic acid directly promotes embryo maturation, with optimal concentrations varying by species and genotype. For example, *Picea abies* (Norway spruce) matures best at 30 μM Abscisic acid (ABA), with inhibition occurring at concentrations above 50 μM, while *Pinus sylvestris* (Scots pine) requires higher ABA concentrations of 80–90 μM to achieve the desired maturity [118].

**Table 2:** Chemical parameters requiring optimization in bioreactor-based plant in vitro cultures.

Chemical Parameter	Main Biological Role in Conventional Culture	Specific Relevance in Bioreactor Cultures	Key Optimization Points	References
Sucrose	Carbon source, energy source, and osmotic regulator	Affects sugar depletion, feeding strategy, and the stability of high-density batch cultures	Initial concentration, feeding time, and feeding concentration	[96,98,99]
Salt strength	Provides mineral nutrients and nitrogen sources	Influences osmotic pressure, pH, ion accumulation, and limiting factors during long-term culture	MS, 1/2 MS, B5, WPM, and N/P ratio	[96,106,108]
Plant growth regulators	Regulate proliferation, differentiation, and organ formation	Need to be recalibrated because their effects are influenced by liquid immersion, aeration, shear stress, and bioreactor configuration	Type, concentration, and stage of addition	[64,113,116]
Elicitors	Activate defense responses and secondary metabolism	Influenced by mixing uniformity, timing of addition, and culture growth stage; directly related to overall volumetric productivity	Type, concentration, addition time, and treatment duration	[40,119,120]

#### 4.2.4. Application of Elicitors

The use of elicitors in plant tissue culture is of great research value, especially in plant cell engineering and secondary metabolite production. Elicitors can promote specific physiological responses of plant tissues, such as proliferation, differentiation, and the synthesis of certain specific metabolic products. Elicitors are classified into two main categories, biotic and abiotic; the biotic type mainly includes yeast extract (YE), chitin and chitosan, pectin fragments, bacterial and fungal wall fractions, etc., which mimic pathogen-triggered defenses, and often promote accumulation of phenolpropane pathway products, saponins, lignans, etc., and at the same time, are milder in their effect on growth [121]. Abiotic types, mainly jasmonic acid and methyl jasmonate (JA/MeJA), salicylic acid (SA), ethylene signaling interveners (such as Ag^+^), heavy metals, ultraviolet (UV-B), temperature and osmotic stress, cyclodextrins, nanoparticles, etc., often up-regulate the target’s secondary metabolism significantly, but inhibit growth at doses above the threshold [40]. The timing of the addition of the elicitor has a critical impact on its effectiveness. It is usually recommended to add the elicitor at the end of the logarithmic growth or the beginning of the stabilization phase, when the biomass curve has flattened, the carbon skeleton and the reducing power have already filled up the cell, the cell does not need to compete for the precursors for the proliferation, and the redundant resources can be rapidly diverted to the secondary metabolic pathways. For example, in *Beta vulgaris* suspension cell cultures, the betaine content was increased 1.4-fold compared to the control, despite a decrease in biomass due to the addition of 40 μM MeJA [122]. The production of artemisinin from *Artemisia annua* has been significantly improved in modified stirred tank bioreactors and the addition of an elicitor, methyl jasmonate, to the bioreactor resulted in a further increase in the artemisinin content, with a 2.4-fold increase in the content as compared to that obtained during the incubation period in the bioreactors without the elicitor [36].

The concentration of elicitors used is a key factor in regulating the balance between growth and metabolism, as different concentrations may activate different metabolic pathways. For example, in adventitious root cultures of *Pseudostellaria heterophylla*, 5 mg/L MeJA favored polysaccharide accumulation, whereas 10 mg/L MeJA more effectively promoted the synthesis of total saponins [119]. In addition, biotic elicitors or low doses of abiotic elicitors can sometimes maintain growth while promoting metabolism. For instance, in the cultivation of *Gynura procumbens* (Lour.) Merr. cultures, 0.025% YE not only increased the biomass but also raised the quercetin content by 1.9 times. However, 1 mg/L CuSO_4_ (copper sulfate), although somewhat inhibiting growth, resulted in a significant 13.3-fold increase in kaempferol content [120].

Therefore, when using elicitors to enhance the production of secondary metabolites, it is necessary to consider the type of elicitor, the timing and concentration of the elicitor, and combine it with the specific culture system and the demand of the target products to achieve the best balance between metabolite accumulation and biomass growth [123].

### 4.3. Key Biological and Process Parameters

#### 4.3.1. Inoculation Density

Inoculum density plays a pivotal role in plant cell and tissue culture by triggering or inhibiting colony induction with initial colony size and then reshaping the microenvironment through immediate competition for substrate, dissolved oxygen and space, which directly affects the growth dynamics and efficiency of secondary metabolite synthesis in plant cells and tissues. The optimum range varies between species and culture systems, and generally follows the basic rule that ‘too low a density will retard growth initiation, while too high a density will increase competition for resources and limit mass transfer’ [95]. When the inoculum density is too low, the culture system struggles to accumulate a sufficient amount of intercellular signals within a short time frame, and population sensing is weakened, often leading to delayed growth initiation and ultimately limiting biomass accumulation. On the contrary, over-inoculation will instantly increase cell density, leading to a sharp increase in the demand for nutrient salts and dissolved oxygen in the microenvironment. As micing and mass transfer cannot immediately compensate for this demand, local nutrient deprivation or metabolic waste accumulation may easily occur, thus inhibiting growth and reducing the synthesis of secondary metabolites. This pattern was demonstrated in a variety of culture systems. For example, in the bioreactor culture of *Hypericum perforatum*, increasing the inoculum density did not promote the accumulation of biomass and secondary metabolites, and 3 g/L was proven to be the optimal initial inoculation condition [124,125]. Similarly, in the *Gynura procumbens* adventitious root system, an inoculation density of 3 g/L resulted in the highest biomass, growth rate, and phenolic and flavonoid contents, while further increasing the density led to declines in all parameters [77].

Therefore, determining the appropriate inoculum density is critical during bioreactor process development. A balance needs to be found between promoting rapid growth initiation and avoiding excessive competition in order to maximize biomass and target metabolite productivity simultaneously.

#### 4.3.2. Culture Period and Harvesting

Inoculum density is a key factor affecting the total yield and accumulation of bioactive compounds under in vitro plant culture conditions, and the optimal inoculum density is influenced by plant species, cell line, nutrient composition, medium conditioning and production scale. Plant growth in bioreactors follows a pattern of late, logarithmic, steady and decline phases. To increase productivity, harvesting needs to be done at specific stages of the growth curve based on the accumulation pattern of the target product (biomass or metabolite).

If the goal is to obtain maximum biomass, harvesting should be done at the end of logarithmic growth or early in the stabilization period. This is the time of high cell density and physiological activity. In batch culture, for example, most suspension cells can reach this state within 20 to 30 days [126]. For secondary metabolites that require induction, the peak of synthesis often lags behind biomass growth. For this reason, elicitors such as methyl jasmonate can be added when cell growth is close to the stabilization phase, and intensive monitoring and timely harvesting can be carried out within 2 to 10 days after treatment to capture the peak of product accumulation and avoid yield decline in the later stages [127,128,129]. For instance, in *Mentha* suspension cells, MeJA pulsing led to a rapid increase in rosmarinic acid, which was complete within 48 h [130]. Batch cultures are simple to operate but have rapid nutrient depletion and short production cycles.

Replenishment batch culture, on the other hand, allows for supplementation of critical nutrients during the culture process and delays growth arrest, thus extending the time for efficient production and increasing the total yield. This method has been widely used in the field of recombinant proteins [131].

For organotrophic culture systems such as hairy roots and multiple shoots, mass transfer to the core region is often limited during the late growth phase due to dense tissue entanglement, and local necrosis may even be triggered. Therefore, timely harvesting at the end of the rising biomass phase or the use of repetitive batch culture strategies can help to maintain high volume-time yields [132,133]. In temporary submerged bioreactors, the regulation of the culture cycle is further reflected in the ‘submergence and drainage’. Optimizing the frequency and duration of submergence is the key to balancing tissue hydration and gas exchange, preventing vitrification, and safeguarding the quality of the sprouts. For example, in *Cannabis sativa* shoot cultures, a 1 min/8 h submergence cycle enhanced biomass accumulation more effectively than a 1 min/4 h cycle [94]. The determination of the optimal culture cycle also needs to be combined with the type of reactor. Stirred tanks facilitate the synchronization of incubation time and nutrient supply through replenishment; air-lifters are better suited to fine-tuning of oxygen supply in long-term incubation; and temporary immersion bioreactors directly regulate the physiological state of tissues through a cycle of alternating immersions [23,134,135,136,137].

Due to the complexity and instability of plant cell cultures, continuous cultures are less commonly used. Strategies such as replenishment batches or repeated batches are more commonly used to maintain high cell viability through cyclic harvesting, thus ensuring stable and efficient product outputs [3].

### 4.4. Interactions of Key Bioreactor Parameters

In bioreactor cultures, it is difficult to adjust only a single parameter to achieve a stable and high yield, and it is necessary to synergistically optimize multiple types of physical, chemical, biological and process parameters. Changes in any one condition may affect other variables. For example, an increase in inoculum density requires a corresponding increase in aeration to maintain dissolved oxygen, and the addition of an elicitor requires a timely adjustment of the harvest time to complete the harvest at the peak of product synthesis. Therefore, multiple parameters must be synergistically optimized to achieve efficient and stable output (Table 3).

In the process development of bioreactors, the role of key parameters and their interactions should be analyzed in an integrated manner through statistical methods such as the design of experiments, in order to determine overall optimal operating conditions, rather than only adjusting single factors independently [82].

## 5. Different Bioreactor Culture Systems for Medicinal Plants

### 5.1. Suspension Cell Culture System

Suspension cell cultures are easy to scale up in bioreactors, but there are two main problems with their application, namely erratic secondary metabolite yields and spontaneous aggregation of cells to form clusters, which hinders material transfer and leads to metabolic heterogeneity within and outside the clusters. To address these issues, two types of bioreactors, mechanically stirred and air-lifted, are currently used: the STR facilitates precise control of kLa, pH, dissolved oxygen, stirring and replenishment; and ‘low shear’ impellers, such as sea paddles or folding paddles, are often chosen for plant cells to reduce tip speed and power input per unit (P/V) [140]. Airlift and bubble column bioreactors rely on air-lift circulation, with little mechanical shear and low energy consumption, and are suitable for ‘shear-sensitive’ suspension cells and pigment efflux, but mixing homogeneity and oxygen transfer in highly viscous culture broths need to be evaluated empirically [141]. The appropriate selection and use of bioreactors can significantly improve culture outcomes. For example, in vitro callus cultures of *Rhodiola* grew poorly and appeared chlorotic in shake flasks, but remained green and vigorous in an airlift bioreactor. After 25 days of culture, the biomass yield in the bioreactor was significantly higher than that in the shake flasks, representing a 2.8-fold increase [14,78]. The key basis for achieving efficient production lies in obtaining cell lines with excellent traits. Selection of cell lines with rapid growth and stable metabolite synthesis capacity by means of mutagenesis screening, clump cloning or metabolic engineering is an important prerequisite for the success of the process. Proven potential of suspension cell culture in the industrialization of medicinal ingredients. The large-scale production route for *paclitaxel*, for example, is based on large STRs, where process scale-up was systematically optimized around the oxygen transfer rate (OTR) and inlet gas composition, ultimately pushing taxane-type compounds toward commercial realization [140]. *Lithospermum erythrorhizon* is a classic example of the industrial production of secondary metabolites via plant cell culture. Fujita et al. [142,143] established a large-scale production system for shikonin derivatives using suspension cell culture in a stirred-tank bioreactor. Through medium optimization and enhanced oxygen supply, the yield of shikonin derivatives reached 1400–2300 mg/L. Subsequent industrialization studies showed that the shikonin yield obtained from a 750 L stirred-tank bioreactor containing 600 L of culture medium within approximately two weeks was equivalent to the total yield from 17.6 hectares of field cultivation over four years, demonstrating the significant advantages of plant cell bioreactors in terms of production efficiency and land utilization [144]. In the case of *Catharanthus roseus*, which produces indole alkaloids such as ajmalicine and catharanthine, a 20 L airlift bioreactor achieved 323 µg g^−1^ DW ajmalicine under cost-optimized conditions (using tap water and sucrose). In a 30 L stirred bioreactor, the timely addition of ABA yielded 85 mg L^−1^ of catharanthine within 10 days, and further modification of the sparging gas composition influenced the formation ratio of ajmalicine to serpentine [145]. Similarly, suspension culture studies of artemisinin [146], lignans from *Schisandra sphenanthera*, and phenolic compounds from Verbena species [147] have demonstrated significantly higher yields than conventional cultivation. The magnitude of the difference is sufficient to cover the cost of process conversion, further validating the generalizability and feasibility of this technology pathway in the production of multiple active ingredients [33] (Table 4).

### 5.2. Organ Culture Systems

Adventitious Roots (AR) are sensitive to shear and easily entangle. Bioreactors can provide uniform oxygen and nutrients under low shear, far better than shaking flasks and static culture, and have been scaled up to the level of 100 liters–kiloliters [148]. Many root actives (e.g., hypericin, gentiopicroside, baicalin, withanolides, etc.) are higher and more stable in the AR system and can be further enhanced by inducing agents. Furthermore, these yields can be further enhanced through the application of elicitors, demonstrating the robustness and scalability of AR systems for industrial production of plant-derived metabolites [22,149]. Compared with suspension cells, adventitious roots possess a complete organ structure, and their secondary metabolic profile is closer to that of natural roots. Airlift and bubble column bioreactors, characterized by pneumatic agitation, low shear stress, and minimal mechanical damage to roots, are suitable for volumetric scale-up and continuous aeration of fibrous adventitious roots (AR) [150]. Stirred-tank bioreactors, on the other hand, facilitate online monitoring and nutrient feeding but require low impeller speeds and modified impeller designs to reduce shear stress, and are therefore commonly used for pilot-scale cultures or shear-tolerant materials. Studies have shown that, in *Panax ginseng* adventitious root cultures, the optimal reactor type for root growth is the 120° conical bubble column bioreactor. The growth of *P. ginseng* adventitious roots in this reactor followed a typical sigmoidal (“S-shaped”) curve, reaching the maximum biomass on day 40. At this point, the fresh weight, dry weight, and dry weight multiplication ratio reached their highest values—113.15 g, 9.62 g, and 63.13-fold, respectively [53,151]. Xiang cultured *Acanthopanax senticosus* adventitious roots in a 10 L airlift bioreactor for approximately 30 days, during which the biomass increased by about 40-fold. Under induction with 100 μmol/L MeJA, the total triterpenoid saponin content reached 2.3 times that of the control group, while the expression levels of key enzyme genes in the triterpenoid biosynthetic pathway—SQS, SQE, BAS, P450, and UGT—were elevated to 1.3–58.6 times higher than those of the control [152]. In *Rehmannia glutinosa* adventitious root cultures, scale-up in balloon-type air-lift bubble bioreactors, combined with methyl jasmonate elicitation, enhanced biomass accumulation and acteoside production. In particular, the 20 L bioreactor produced adventitious roots with higher biomass, acteoside content, and antioxidant activity than shake-flask cultures, while FT-NIR analysis indicated no significant difference in the overall chemical composition between the in vitro-derived roots and commercial samples [153]. Studies have also demonstrated that the air-bag bubble bioreactor is suitable for the large-scale commercial production of adventitious roots of woody medicinal plants such as *Eurycoma longifolia* [154] (Table 5).

Hairy root systems rely on genetic transformation of plant tissues by *Agrobacterium rhizogenes* Ri plasmids, whose verified genetic and biosynthetic stability provides the foundation for establishing standardized parameters that influence biomass accumulation and the production of bioactive compounds [39]. In the actual cultivation process of this system, the root segments will be entangled with each other to form dense clusters, the void ratio plummets, the resistance to diffusion of dissolved oxygen and nutrients increases steeply, the metabolic field inside the clusters appears to be significantly differentiated, the product synthesis area and the consumption area spatial mismatch, and the contradiction is even more prominent after amplification. Airlift and bubble column bioreactors, which are suitable for most hairy root cultures due to their low shear and high gas–liquid contact area, can be operated in balance with thinning of clusters by increasing kLa while avoiding root clusters that are too dense to cause hypoxia through the apparent gas velocity. Proper adjustment of superficial gas velocity and implementation of root-dispersing operations can effectively balance oxygenation and root density, ensuring stable metabolite production and culture homogeneity [156]. The mechanism of atomized, gas-phase oxygenation reactors, in which the roots are exposed to the oxygenated gas phase and nutrients are supplied in droplets or sprays, has a very high oxygenation efficiency and low shear, which is particularly advantageous for dense ‘root mat’ structures [46]. It was found that *Artemisia annua* HR exhibited higher growth and artemisinin yield in an atomized reactor as compared to a bubble column bioreactor [47,157]. Temporary Immersion reactors are designed to prevent hypoxia caused by prolonged liquid immersion by programmed lifting or tilting actions, while also utilizing transient diffusion at the gas–liquid interface to enhance oxygen supply [158,159]. In studies of *Gentiana* spp. Hairy roots, the bubble column bioreactors (BCBs) and the temporary immersion bioreactor (TIS RITA) achieved superior growth compared to shake flasks (EFs) with higher aeration while culturing and continuous air flow through the nebulizer, with most clones exhibiting higher growth parameters. Hairy roots grown in TIS contained the highest amount of flavonoids [160]. The above reactors enhanced the culture efficiency of hairy roots from the perspectives of mixing mechanism, oxygen supply mode and submergence strategy, respectively, and provided reliable technical support for the large-scale cultivation of hairy roots of *Dendrobium* and other medicinal plants. Therefore, rational selection of the reactor and system optimization is a key component in increasing the yield of secondary metabolites from hairy roots [157].

### 5.3. Somatic Embryo and Shoot Culture

Embryo and shoot culture systems are primarily used in medicinal plant biotechnology for efficient micropropagation and the production of specific metabolites. Differentiated somatic embryos and shoots are extremely sensitive to the gas exchange rate within the culture microenvironment. When maintained in static liquid culture beyond a critical period, hypoxic stress and vitrification rapidly intensify, resulting in a sharp decline in proliferation efficiency. The temporary immersion bioreactor is programmed to periodically submerge and drain the culture solution. This forces the cultures to alternate between nutrient uptake and efficient gas exchange at a high frequency. This approach not only prevents vitrification but also produces a uniform culture bed that is amenable to subsequent automated harvesting and subculturing. TIS is a viable alternative to traditional solid media for automated, large-scale production of seedlings and micropotatoes in potato micropotato production systems [64,161,162,163]. In banana micropropagation, a submergence cycle of ‘2 min/4–6 h’ combined with the rational use of cytokinins can significantly increase the proliferation coefficient and seedling survival rate, and at the same time reduce the production cost [164,165,166]. In addition, low-shear airlift reactors, which provide a mild flow field and high dissolved oxygen, represent another viable option for the industrial cultivation of somatic embryos. For instance, somatic embryos of *Santalum album* (East Indian sandalwood) were able to complete their entire developmental process in a 10 L airlift reactor [167]. Similarly, somatic embryos of grape (*Vitis vinifera*) cultured in an airlift system accumulated 4.7-fold more biomass than those on solid medium, while also effectively promoting resveratrol biosynthesis [168]. In a bubble column reactor, Siberian ginseng (*Eleutherococcus senticosus*) embryos exhibited not only a higher germination conversion rate but also the induction of secondary somatic embryos. Additionally, they showed elevated proline content and antioxidant enzyme activities compared to those in suspension culture [169]. The above cases show that, whether temporary immersion or air-lift driven, bioreactors have liberated somatic embryos and buds from manual Petri dishes and transformed them into programmable, scalable, and online-monitored production units, and the bioreactor systems constructed based on different principles, such as emporary immersion and air-lift driven, have provided practical technological support for the large-scale propagation of somatic embryos and buds and the high-efficiency production of related metabolites.

Overall, suspension cell culture is more suitable for stirred-tank or airlift bioreactors with high mass-transfer efficiency and strong online control capability, and is particularly appropriate for the large-scale production of high-value metabolites such as shikonin, paclitaxel, and indole alkaloids. Adventitious roots and hairy roots possess relatively intact organ structures and metabolic characteristics closer to those of natural roots; however, they are sensitive to shear stress and prone to entanglement, and are therefore more suitable for airlift, bubble column, balloon-type bubble, spray, or temporary immersion bioreactors. Details are provided in Supplementary Table S1, which summarizes representative production cases of medicinal plant suspension cells, adventitious roots, and hairy roots in different bioreactor systems. These cases indicate that bioreactor type, culture morphology, aeration and oxygen supply, shear stress, feeding strategy, and elicitor treatment jointly determine biomass accumulation and the production of characteristic metabolites.

## 6. Feasibility and Commercial Application of Bioreactor-Based Production of Medicinal Plants

Bioreactor technology provides an important engineering platform for the controllable culture of medicinal plant cells, tissues, and organs, offering significant advantages in shortening production cycles, improving environmental controllability, reducing seasonal and geographical constraints, and enhancing product quality consistency. Compared with traditional field cultivation, this technology is particularly suitable for the development of medicinal plant resources that are naturally scarce, require long field harvesting periods, have low active ingredient content, or exhibit quality that is highly susceptible to environmental influences. Previous studies have shown that various medicinal plant cell, hairy root, and adventitious root culture systems demonstrate promising potential for bioreactor culture [1,40,91]. However, bioreactors are not a complete substitute for traditional cultivation. Their commercial value depends on multiple factors, including the market value of the target product, the supply stability of natural sources, the productivity of the culture system, downstream extraction costs, and quality control requirements. For medicinal plants that have low field cultivation costs, stable supply, and mature extraction processes, bioreactor systems may not necessarily achieve significant economic advantages in the short term [1,170]. To further compare the differences in characteristic metabolite production between field-cultivated medicinal plants and bioreactor culture systems, this review summarizes the relevant information in Supplementary Table S2.

From a commercial application perspective, the production of paclitaxel via plant cell culture represents one of the most representative success stories to date. Suspension cultures of *Taxus* cells have been employed for the commercial production of paclitaxel and other taxane drug precursors, demonstrating that plant cell bioreactors possess practical commercial feasibility for the production of high-value, low-natural-abundance active ingredients with clear market demand [33,171]. In addition, adventitious root culture of ginseng has also been systematically studied in large-scale bioreactors and is considered to have application potential for producing ginsenosides and functional ingredients [172]. However, these cases also indicate that successful industrialization does not rely solely on culture growth rate or target compound content, but also depends on the establishment of stable cell lines or root materials, scalable low-shear reactor design, batch-to-batch consistency control, downstream recovery efficiency, and the market value of the final product [23,173]. It is worth noting that the number of plant cell or organ culture cases that have truly achieved stable commercial production remains relatively limited. Success stories such as paclitaxel more likely represent the feasibility of high-value pharmaceutical ingredients under specific process conditions and cannot be simply generalized to imply that all medicinal plant culture systems possess industrial feasibility.

From an industrial scale-up perspective, bioreactor-based production of medicinal plants still faces significant engineering and biological limitations. Plant cells, hairy roots, and adventitious root cultures typically exhibit high shear sensitivity and structural heterogeneity. During scale-up, problems such as cell aggregation, root entanglement, localized hypoxia, nutrient gradients, uneven mixing, and metabolic fluctuations are prone to occur [1,91,170]. As the culture volume increases, oxygen transfer efficiency, mixing time, and shear force distribution no longer maintain a linear relationship with those observed in small-scale systems. Consequently, high-yield results obtained under laboratory conditions are often difficult to directly translate into pilot-scale or commercial production [174]. Therefore, the scale-up of bioreactors for medicinal plants should not merely aim at increasing volume, but should take maintaining physiological stability, metabolic flux stability, and batch-to-batch consistency of the culture as the core evaluation criteria. Airlift reactors, wave-mixed disposable bioreactors, temporary immersion bioreactors, and specialized reactors suitable for root culture offer certain advantages in reducing mechanical damage and improving mass transfer; however, their applicability still requires systematic validation based on the culture object, product type, and production scale.

Cost control and downstream processing are key factors determining whether this technology can be successfully industrialized. The comprehensive cost of bioreactor-based production of medicinal plants includes not only reactor equipment, culture media, energy, sterilization, labor, and equipment depreciation, but also losses due to contamination, quality testing, waste liquid treatment, as well as extraction and purification steps [175]. In many culture systems, plant secondary metabolites are present at low concentrations and often co-exist with polysaccharides, proteins, phenolic compounds, and cell wall components, leading to complex extraction, separation, and purification processes, as well as significantly increased costs. In some systems, downstream processing costs may even become the primary factor limiting commercialization [170,173]. Therefore, future process development should not focus solely on biomass accumulation or the increase in a single product content during the upstream cultivation stage, but should also integrate product release mode, in situ adsorption, two-phase culture, continuous recovery, extraction yield, and purification grade into the overall evaluation. For example, in the plant cell fermentation process for paclitaxel, resin adsorption can be used to capture the target product from the culture system, thereby reducing the burden of subsequent purification [176]. If the target product can be secreted into the culture medium or can be recovered in situ via methods such as resin adsorption or membrane separation, it would be more favorable for reducing downstream processing difficulty and enhancing industrial feasibility.

When the target product is positioned as a pharmaceutical ingredient or is intended to enter the high-standard functional ingredient market, compliance with GMP or equivalent quality management system requirements also becomes an unavoidable challenge for the commercialization of bioreactor technology [177]. Compared with laboratory-scale research, industrial-scale production requires the establishment of a complete quality management system encompassing germplasm origin, preservation of cell lines or root materials, seed bank construction, culture process monitoring, batch record-keeping, contamination control, genetic stability testing, quantification of target compounds, impurity profiling, and final product release. For products derived from medicinal plants, additional attention should be paid to issues such as the fingerprint profile of active ingredients, pesticide and heavy metal residues, endotoxin or microbial limits, medium residues, and batch-to-batch consistency [178,179,180,181]. Currently, many studies remain at the laboratory or pilot scale, with insufficient reporting on long-term continuous subculture stability, batch repeatability, cost structure, and quality release standards. This limits their translation into commercial production [1,91,170].

## 7. Conclusions

Bioreactor technology for medicinal plants provides an important platform for the conservation of rare and endangered medicinal resources, the production of high-value-added active ingredients, and the stabilization of medicinal material quality. This review systematically summarizes the application progress of plant cell, hairy root, adventitious root, and temporary immersion culture systems in different types of bioreactors and further analyzes the key engineering and biological factors affecting large-scale production. Overall, this technology has the potential to shorten production cycles, improve environmental controllability, and enhance product quality consistency. However, its industrial application remains limited by factors such as scale-up stability, metabolic fluctuations, downstream processing costs, GMP quality control, and commercial feasibility.

Therefore, future research on medicinal plant bioreactors should shift from solely pursuing biomass or target product content toward integrated process development that is scalable, controllable, verifiable, and economically feasible. On the one hand, it is necessary to establish scale-up criteria suitable for plant cell and organ culture by integrating fluid dynamics, process analytical technology, online sensing, and intelligent control. On the other hand, the stable biosynthetic capacity of target products should be enhanced through metabolic engineering, elicitor optimization, precursor feeding, and screening of high-yield cell lines. Meanwhile, techno-economic analysis and life cycle assessment should be strengthened, incorporating indicators such as unit product cost, energy consumption, equipment depreciation, contamination loss rate, and downstream yield into the evaluation system, and systematically comparing them with pathways including field cultivation, wild collection, and chemical synthesis. Only when a balance is achieved among productivity, quality, stability, regulatory compliance, and cost control can bioreactor technology more effectively serve the sustainable utilization of medicinal plant resources and the industrial production of active ingredients.

## Figures and Tables

**Table 1:** Comparison of Major Bioreactor Types in Engineering Characteristics and Applicability.

Reactor Type	Mixing/Oxygen Transfer Mechanism	Shear Force Level	Suitable Tissue Type	Advantages	Disadvantages	References
Stirred Tank	Mechanical stirring	High	Suspension cells	High mass transfer efficiency, easy process control and scale-up	High shear force, prone to damaging sensitive tissues	[35]
Wave-mixed	Platform rocking to generate waves	Low	Suspension cells, hairy roots, and adventitious roots	Low shear, single-use design prevents cross-contamination	Limited online monitoring and restricted scalability	[62]
Bubble Column	Gas bubbling	Low	Adventitious roots, somatic embryos, and buds	Simple structure with extremely low shear force	Low mixing efficiency, with potential dead zones	[63]
Sprinkle or Spray	Nutrient mist spraying	Extremely Low	Hairy roots, somatic embryos, and buds	High gas-phase oxygen supply efficiency and extremely low shear	Requires high atomization uniformity, a complex system	[46]
Temporary	Periodic liquid immersion	Low	Buds, somatic embryos, and micropropagation	Balances nutrient absorption and gas exchange, with high automation potential	Immersion cycles need optimization, prone to vitrification	[64]
Airlift	Gas bubbling–driven circulation	Medium-Low	Suspension cells, adventitious roots, and hairy roots	Relatively low shear force, simple structure, and easy to scale up	Uneven mixing under high-density culture conditions	[44]

Note: Both the airlift and the bubble column belong to the pneumatic bioreactors.

**Table 3:** Optimization objectives and synergistic strategies for key bioreactor parameters.

Parameter Category	Optimization Objective	Common Optimization Strategies	Synergistic Relationships with Other Parameters	Interactions with Other Parameters	References
Physical parameters	Agitation speed	Balance oxygen transfer (kLa) and shear stress.	“Large impeller with low rotational speed” to reduce tip speed; using kLa as the evaluation index.	Together with the aeration rate, it determines kLa; high-density cultures require increased agitation speed or aeration rate.	[70,138]
	Aeration rate	Maintain dissolved oxygen while suppressing foaming and shear damage.	Identify the species-specific optimal vvm range.	Affects dissolved oxygen and shear stress; higher agitation speed may reduce dependence on high aeration.	[139]
Chemical parameters	Medium composition	Balance nutrient supply with osmotic pressure and signaling regulation.	Optimize the concentration and ratio of carbon sources and ionic strength; apply a fed-batch strategy.	Sucrose concentration influences osmotic pressure and interacts with ionic strength; nutrient consumption rate determines the timing of feeding.	[95,96]
	Growth regulators	Adapt to reactor conditions with precise developmental control.	Re-optimize concentrations and combinations based on reactor conditions.	Interacts with elicitors through signaling pathways; cytokinin concentration affects vitrification level, regulated by TIB immersion frequency.	[112,114]
	elicitors	Maximize stress-induced secondary metabolite production.	Add at the late logarithmic phase; precisely control the concentration to balance growth inhibition and metabolic promotion.	The timing of addition depends on the growth phase; its effect is influenced by the nutrient status of the basal medium.	[40,122,123]
Biological and process parameters	Inoculation density	Achieve rapid initiation while avoiding excessive competition.	Determine the optimal inoculation density range.	High inoculation density requires greater aeration and nutrient supply; it affects the duration of the culture cycle.	[95]
	Culture duration and harvesting	Harvest at the peak of growth and metabolic activity.	Define based on growth curves and product synthesis kinetics; extend the production phase through a fed-batch cultivation strategy.	Determined by growth rate, nutrient consumption, and elicitor response; reactor type influences cycle design.	[3]

**Table 4:** Summary of reactor selection and strategies for suspension cell culture.

Reactor Type	Core Advantages	Application Scenarios	References
Stirred Tank	High mass transfer efficiency, precise process control, and easy scale-up	For systems insensitive to shear and requiring complex feeding or process analysis	[35]
Airlift	Extremely low shear force, simple structure, and low energy consumption	For shear-sensitive cell lines with easily secreted products	[44]
Wave-mixed	Low shear, single-use design prevents cross-contamination	Early-stage process development and small- to medium-scale pilot production	[62]

**Table 5:** Comparison of bioreactor types suitable for adventitious root cultivation.

Reactor Type	Core Advantages	Application Scenarios	References
Airlift/Bubble Column	Extremely low shear force, relatively simple structure, suitable for scale-up	Large-scale production (hundreds to thousands of liters)	[63]
Spherical Bubble	Milder flow field reduces wall adhesion	Optimization of metabolite synthesis	[51,52,53]
Wave-mixed	Single-use design prevents cross-contamination	Pilot-scale process development	[62]
Stirred type	Facilitates online monitoring and nutrient feeding	Small- to medium-scale cultures requiring precise shear control	[155]

## Data Availability

No datasets were generated or analyzed during the current study.

## Supplementary Materials

The following supporting information can be downloaded at https://www.mdpi.com/article/10.3390/biology15131025/s1: Table S1: Representative cases of biomass and target metabolite production in medicinal plant bioreactor cultures; Table S2: Comparison of characteristic metabolite production between field-cultivated medicinal plants and bioreactor culture systems.
