# Supplementary material for: Bioreactor Technology for Medicinal Plant In Vitro Cultures: Systems, Applications, and Future Perspectives
Source: Biology (Basel). 2026 Jun 27;15(13):1025. doi: 10.3390/biology15131025 (PMC13360316; doi:10.3390/biology15131025)
Supplement: Supplementary file 1 [file biology-15-01025-s001.zip › biology-4352015-supplementary.pdf]

Table S1. Representative cases of biomass and target metabolite production in medicinal plant bioreactor cultures

Plant species	Culture type	Bioreactor type	Scale/conditions	Target compounds	Main production results	Main limitations	References
Lithospermum erythrorhizon	Suspension cell culture	Stirred-tank reactor	750-L culture tank, 600 L culture medium, approximately 2 weeks	Shikonin	The cell culture cycle was short, and shikonin content and time-based productivity were significantly higher than those of traditional cultivation.	Stability of high-producing cell lines, cell aggregation, and mass-transfer control	[141-144]
Taxus spp.	Suspension cell culture	Stirred-tank reactor	Large-scale industrial culture	Paclitaxel / taxanes	A commercial production route has been established.	Cell-line screening, shear stress, feeding, and elicitation regulation	[127,128,140,]
Catharanthus roseus	Suspension cell culture	Airlift bioreactor	20 L	Ajmalicine	Indole alkaloids can accumulate in bioreactors.	Aeration, gas composition, and timing of elicitor addition	[145]
Catharanthus roseus	Hairy roots	Modified bubble column reactor, PUF foam support	5-L reactor with a 3-L working volume; inoculum density of 0.5 g/L DW; 0.3 vvm; 30 days	Ajmalicine	Biomass reached 7.7 ± 1.1 g/L DW, ajmalicine production reached 34 ± 2.3 mg/L, and volumetric productivity was 1.13 mg/L/day, outperforming conventional bubble column and rotating drum reactors.	Hairy roots are shear-sensitive and prone to entanglement; therefore, agitation, aeration, and elicitation conditions need to be optimized.	[79]

Rhodiola spp.	Callus / cell culture	Bioreactor; airlift bioreactor	Optimization of aeration rate	Salidroside	Biomass and salidroside accumulation were significantly affected by aeration rate.	Balancing biomass growth and product accumulation	[78]
Artemisia annua	Hairy roots	STR / mist bioreactor	Fed-batch culture and MeJA elicitation	Artemisinin	MeJA elicitation and feeding strategies can enhance artemisinin accumulation.	Hairy roots are shear-sensitive and are easily affected by aeration and agitation.	[36,102,157]
Arachis hypogaea	Hairy roots	Mist bioreactor	1-L and 20-L scales	Biomass	Dry weight reached 12.75 g DW/L at 1 L and 7.77 g DW/L after scale-up to 20 L.	Mist uniformity, droplet deposition, and scale-up effects	[47]
Panax ginseng	Adventitious roots	Balloon-type bubble bioreactor	5 L, 20 L, and 500 L	Ginsenosides and biomass	The 500-L system produced approximately 74.8 kg of roots, and ginsenoside levels were similar to those of field-grown roots.	Root clumping, oxygen-supply uniformity, and equipment cost	[52,53]
Panax ginseng	Adventitious roots	120° conical bubble bioreactor	Day 40	Biomass and ginsenosides	Fresh weight, dry weight, and proliferation rate all reached relatively high levels.	Bioreactor configuration and inoculum density affected root distribution.	[151]
Oplopanax elatus	Adventitious roots	10-L airlift bioreactor	Approximately 30 days; 100 µmol/L MeJA	Total triterpenoid saponins	Adventitious-root biomass increased approximately 40-fold, and total triterpenoid saponins after MeJA elicitation were 2.3	MeJA concentration and timing of addition need to be optimized.	[152]

					times those of the control.		
Rehmannia glutinosa	Adventitious roots	Balloon-type bubble bioreactor	Compared with shake-flask culture	Acteoside	Adventitious root biomass, acteoside content, and antioxidant activity were all higher than those in shake-flask cultures	Root density, mass transfer, and low-shear oxygen supply	[153]
Gynura procumbens	Adventitious roots	Aeration-controlled reactor system; balloon-type bubble bioreactor	0.15 vvm; elicitor treatment	Flavonoids, quercetin, and kaempferol	An aeration rate of 0.15 vvm favored biomass and flavonoid accumulation; yeast extract (YE) and $CuSO_4$ increased flavonoid compounds.	Excessive aeration or excessive elicitor levels may inhibit growth.	[77]
Gentiana spp.	Hairy roots	BCB / TIB	Compared with shake-flask culture	Flavonoids	Hairy roots in the TIB showed higher flavonoid content.	Hairy-root entanglement, immersion frequency, and oxygen-supply control	[160]

Table S2. Comparison of characteristic metabolite production between field-cultivated medicinal plants and bioreactor culture systems

Plant species	Target compounds	Advantages of bioreactor culture	Stability of active ingredients	Stability of active ingredients	Potential for large-scale production	Complementary significance to field cultivation	References
Lithospermum erythrorhizon	Shikonin	Slow accumulation of root-derived products, long cultivation cycles in traditional field cultivation, and large land requirements.	Short culture cycle of cell culture, suitable for the production of high-value pigment compounds	Relatively high; can be controlled through cell-line selection and optimization of culture conditions	High; classical studies have established a strong basis for shikonin production by cell culture	Can serve as an important alternative or supplement for industrial shikonin production	[14,141]
Taxus spp.	Paclitaxel / Taxanes	Slow plant growth, limited resources, and long harvesting cycles.	Suspension cell culture can overcome the limitation of tree resources and be used for taxane	Relatively high, but dependent on stable high-yielding cell lines and elicitation	High; scale-up studies and a basis for commercialization have been established for cell suspension culture	Can reduce dependence on natural Taxus resources	[127,128,140]

			production	strategies			
Panax ginseng	Ginsenosides	Long cultivation cycles, with quality affected by geographical origin, cultivation age, soil conditions, and diseases.	Adventitious root culture has a short culture cycle, and root culture more closely resembles the metabolism of natural roots	Relatively high; saponin levels can approach those in field-grown roots	High; pilot-scale and scale-up cultivation cases have been reported for adventitious roots	Can serve as a standardized raw material source for ginsenosides	[52,53,151]
Oplopanax elatus	Triterpenoid saponins	Root-derived medicinal materials face resource constraints and have a long production cycle.	Adventitious roots can be rapidly propagated, and MeJA can enhance triterpenoid saponin biosynthesis	Relatively high; can be regulated by elicitors	Moderate to high; 10-L air-lift bioreactor cases have been reported	Suitable for supplementary production of rare or slow-growing root medicinal materials	[148,152]
Rehmannia glutinosa	acteoside	The composition is influenced by variety,	Adventitious root bioreactors can enhance	Relatively high; closed cultivation	Moderate to high; studies on scale-up cultivation and	Can serve as a supplementary source of raw	[153]

		geographical origin, and cultivation conditions.	acteoside accumulation	facilitates batch-to-batch stability	elicitation-enhanced metabolite accumulation have been reported	materials rich in acteoside	
Artemisia annua	Artemisinin	Artemisinin content in field-grown plants is affected by cultivar, environmental conditions, and harvest time.	Hairy root bioreactor systems can enhance artemisinin production through feeding and MeJA elicitation	Moderate to high, but shear stress, oxygen supply, and feeding conditions need to be controlled	Moderate to high; studies using STR and gas or liquid-phase bioreactors have been reported	Can supplement field-based artemisinin supply, but further scale-up validation is still needed	[36,102,157]
Catharanthus roseus	Ajmalicine and other indole alkaloids	The content of target alkaloids is low and is affected by plant developmental stage and environmental conditions.	Cell or hairy root cultures can be used for the production of high-value, low-abundance alkaloids	Moderate to high; stable culture systems are required	Moderate to high; 20-L air-lift bioreactor and hairy root bioreactor cases have been reported	Can serve as a production platform for rare indole alkaloids	[79,145]

Rhodiola sachalinensis	Salidroside and polysaccharides	Wild or cultivated resources are limited by ecological conditions and growth cycles.	Air-lift bioreactors can promote salidroside and polysaccharide production	Moderate; further systematic comparison with field-grown medicinal materials is required	Moderate; air-lift bioreactor studies have been reported	Can be used for resource conservation and supplementation of active ingredients	[78]
Gynura procumbens	Flavonoids, quercetin, and kaempferol	Flavonoid accumulation is influenced by the growth environment and harvest time.	Aeration rate, inoculum density, and elicitors can directionally enhance flavonoid compounds	Moderate to high, but the effects of elicitors on growth need to be balanced	Moderate; optimization studies of adventitious root bioreactors have been reported	Can serve as a targeted production system for flavonoid compounds	[77,120]
Pseudostellaria heterophylla	Polysaccharides and saponins	Field-grown material quality is affected by geographical origin,	Metabolite accumulation in adventitious roots can be regulated by	Moderate; more direct comparisons with field-grown	Moderate; optimization studies using balloon-type bubble bioreactors	Can be used for target compound-oriented supplementary	[119]

	cultivation practices, and harvest time.	MeJA concentration and bioreactor angle	medicinal materials are needed	have been established	production

Table 2 further compares the differences in characteristic metabolite production between field-cultivated medicinal plants and bioreactor culture systems. Overall, bioreactors are not a simple substitute for field cultivation, but rather transform the production of active ingredients from medicinal plants into a controllable engineering process. For medicinal plants characterized by long growth cycles, scarcity of wild resources, low target compound content, or high susceptibility to environmental influences, bioreactors offer distinct advantages in terms of production cycle, batch-to-batch consistency, contamination control, and preparation of high-value-added constituents. However, for cases relying on intact medicinal plant morphology, complex multi-component synergistic effects, and low-cost supply of bulk medicinal materials, field cultivation remains irreplaceable. Therefore, bioreactors are more suitable as an important complementary platform to field cultivation, serving the purposes of protecting rare and endangered medicinal species, producing standardized active ingredients, and enabling green manufacturing in modern Chinese medicine.
